# Oppositely Charged Nanoparticles Precipitate Not Only at the Point of Overall Electroneutrality

**DOI:** 10.1021/acs.jpclett.3c01857

**Published:** 2023-10-02

**Authors:** Masaki Itatani, Gábor Holló, Dániel Zámbó, Hideyuki Nakanishi, András Deák, István Lagzi

**Affiliations:** † Department of Physics, Institute of Physics, Budapest University of Technology and Economics, Műegyetem rkp. 3, Budapest H-1111, Hungary; ‡ ELKH-BME Condensed Matter Research Group, Műegyetem rkp. 3, Budapest H-1111, Hungary; § Department of Fundamental Microbiology, University of Lausanne, Biophore Building, CH-1015 Lausanne, Switzerland; ∥ Centre for Energy Research, Institute of Technical Physics and Materials Science, Konkoly-Thege út 29-33, Budapest H-1120, Hungary; ⊥ Department of Macromolecular Science and Engineering, Graduate School of Science and Technology, Kyoto Institute of Technology, Matsugasaki, Sakyo-ku, Kyoto 606-8585, Japan

## Abstract

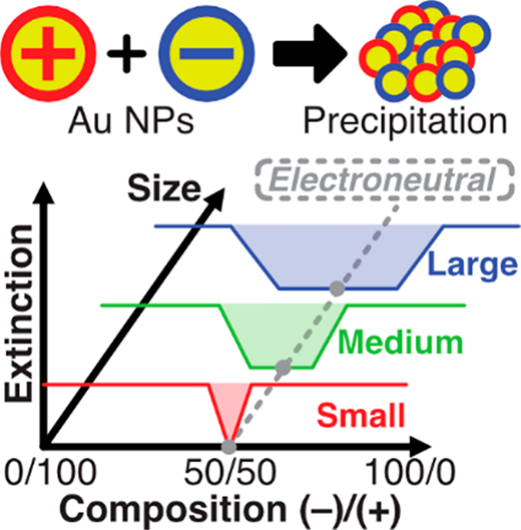

Precipitation of oppositely charged entities is a common phenomenon in nature and laboratories. Precipitation and crystallization of oppositely charged ions are well-studied and understood processes in chemistry. However, much less is known about the precipitation properties of oppositely charged nanoparticles. Recently, it was demonstrated that oppositely charged gold nanoparticles (AuNPs), also called nanoions, decorated with positively or negatively charged thiol groups precipitate only at the point of electroneutrality of the sample (i.e., the charges on the particles are balanced). Here we demonstrate that the precipitation of oppositely AuNPs can occur not only at the point of electroneutrality. The width of the precipitation window depends on the size and concentration of the nanoparticles. This behavior can be explained by the aggregation of partially stabilized clusters reaching the critical size for their sedimentation in the gravitational field.

Understanding the self-assembly of nanoscopic building blocks is one of the essential challenges in nanoscience and nanotechnology.^1−5^ Answering the related questions raised is important not only from the fundamental but also from the applied science point of view. Untangling the general processes and exploring the main interactions existing between the building blocks allow us to control and drive the assembly, generating various hierarchical and higher-order structures.^6−9^

There are several governing forces at the nanoscale that order the self-assembly. Among them, two interactions, the attractive van der Waals (VdW) and electric double-layer interactions, are dominant.^10−13^ Competition between these two interactions can generate various equilibrium and out-of-equilibrium structures ranging from one to three dimensions.^14−18^ These two interactions regulate the aggregation and precipitation of the oppositely charged nanoparticles (NPs) as well.^19^ Precipitation, including nucleation and growth processes from ions, is a well-understood phenomenon from the thermodynamic as well as kinetic points of view.^20−23^ However, much less is known about the precipitation of oppositely charged spherical nanoparticles (NPs), also called nanoions.

Previous studies in the past two decades showed that oppositely charged NPs precipitate rapidly only at the point of electroneutrality, wherein their charges are macroscopically compensated.^24−31^ Later this empirical statement was extended with the statement that the oppositely charged NPs exhibit this behavior at the point of overall electroneutrality only if the concentration of NPs exceeds a threshold concentration (termed precipitation threshold concentration).^32^

It has been shown earlier that very small (a few nanometers in diameter), oppositely charged nanoparticles show ionic-like behavior during their aggregation, and large, ordered nanoparticle crystals are formed at the point of the overall electroneutrality, where the aggregate formation can be qualitatively described considering the free-energy change associated with the ordered nanoparticle-crystal formation.^33^ As for these “nanoions”, the electric double-layer interaction has an effective range comparable to that of the particle diameter. The solubility of the precipitate of nanoions can be considered to be zero, and when one polarity of nanoions is in excess, the formation of core–shell structures can be anticipated; that is, the minority component is shielded by the majority of NPs, resulting in small nanoparticle clusters that can remain stable over time without significant sedimentation.^24^ For larger particle diameters, similar behavior can be expected, but as their concentrations become similar, an earlier onset of sedimentation might occur.

Here we show that this empirical law of the precipitation of oppositely charged NPs should be extended further with the fact that the precipitation occurs not only at the point of the electroneutrality, but rather there is a certain precipitation window around the electroneutrality, enabling the particle–particle assembly. The width of this precipitation window depends on the size of the NPs and the concentration of the NP solutions. In our study, we follow the definition suggested by the International Union of Pure and Applied Chemistry (IUPAC) for chemical precipitation, namely, precipitation is the process in which a solid material sediments from a liquid solution.^34^

To investigate the precipitation behavior of oppositely charged AuNPs, we used three different sizes of AuNPs (2.2, 4.6, and 9.1 nm) and sample concentrations (0.56 and 0.26 mM in terms of gold atoms, corresponding to extinction values at the peak of the surface plasmon resonance of 1.7 and 0.8, respectively). Before the precipitation experiments, we verified that the precipitation point when a rapid (on the time scale of several ten seconds) precipitation occurred—expressed as a ratio of the amount of the negatively (*n*_–_) and all charged (*n*_–_ + *n*_+_) NPs (χ = *n*_–_/(*n*_–_+ *n*_+_))—was close to 0.5 within an experimental error of 5% by an electrostatic titration using the solutions of oppositely charged AuNPs of 0.56 mM. This procedure was described in detail in our previous study.^32^ Our finding indicated that the numbers of positively and negatively charged thiols attached to the surfaces of AuNPs were equal on average, which is consistent with the findings published in other studies.^24,32^ The details of the experiments can be found in the Supporting Information.

In the experiments (Figure 1), two solutions of oppositely charged AuNPs with various volume ratios were mixed in a plastic cuvette, keeping the overall particle concentration constant. This procedure differed from the method used in previous studies in which the authors titrated the solution of one polarity with the solution of oppositely charged AuNPs.^24,25^ Whereas the individual samples are stable, both the electric double layer and dispersion interactions are attractive between the oppositely charged particle types, resulting in a net attractive interaction on the order of several tens of *kT* (Figure S1) that leads to their rapid heteroaggregation.^35^ It has to be emphasized that the applied volume ratio also corresponds to the ratio of the total charges introduced into the samples. After mixing, the solutions were left undisturbed at room temperature. After 1 h, the extinction of the samples was measured by UV–vis spectrophotometry, and the values were extracted at λ = 523 nm, corresponding to the initial plasmon resonance peak as well as at λ = 400 nm, where the absorption is solely determined by the interband transitions in gold and hence can be used to assess the amount of Au^0^ in the light path.^36−38^ We chose 1 h for the precipitation experiments because it was reported that the electrostatic precipitation occurred in a few ten seconds at the point of electroneutrality.^24,29,32^ Therefore, 1 h (which is around 2 orders of magnitude greater than the time scale of the precipitation at the point of electroneutrality) was a reasonable choice to investigate the colloidal stability of the samples. The precipitation consists of two distinct, consecutive, and well-separated steps that can be easily followed by UV–vis spectroscopy. The first step is the aggregation of AuNPs into clusters, which can be resolved by a red-shift in the spectrum and an increase of the peak of the extinction, consistent with the plasmon coupling upon particle clustering.^39^ The cluster growth or eventual cluster-to-cluster aggregation leads to the loss of dispersion stability, which is manifested in the sedimentation of the sample. Further, these clusters lose their colloidal stability upon being merged into larger clusters and sediment from the solution. This process leads to a decreasing sample extinction. In other words, the formation of clusters and their destabilization (sedimentation) can be monitored and distinguished by measuring the extinction of the samples.

**Figure 1:**
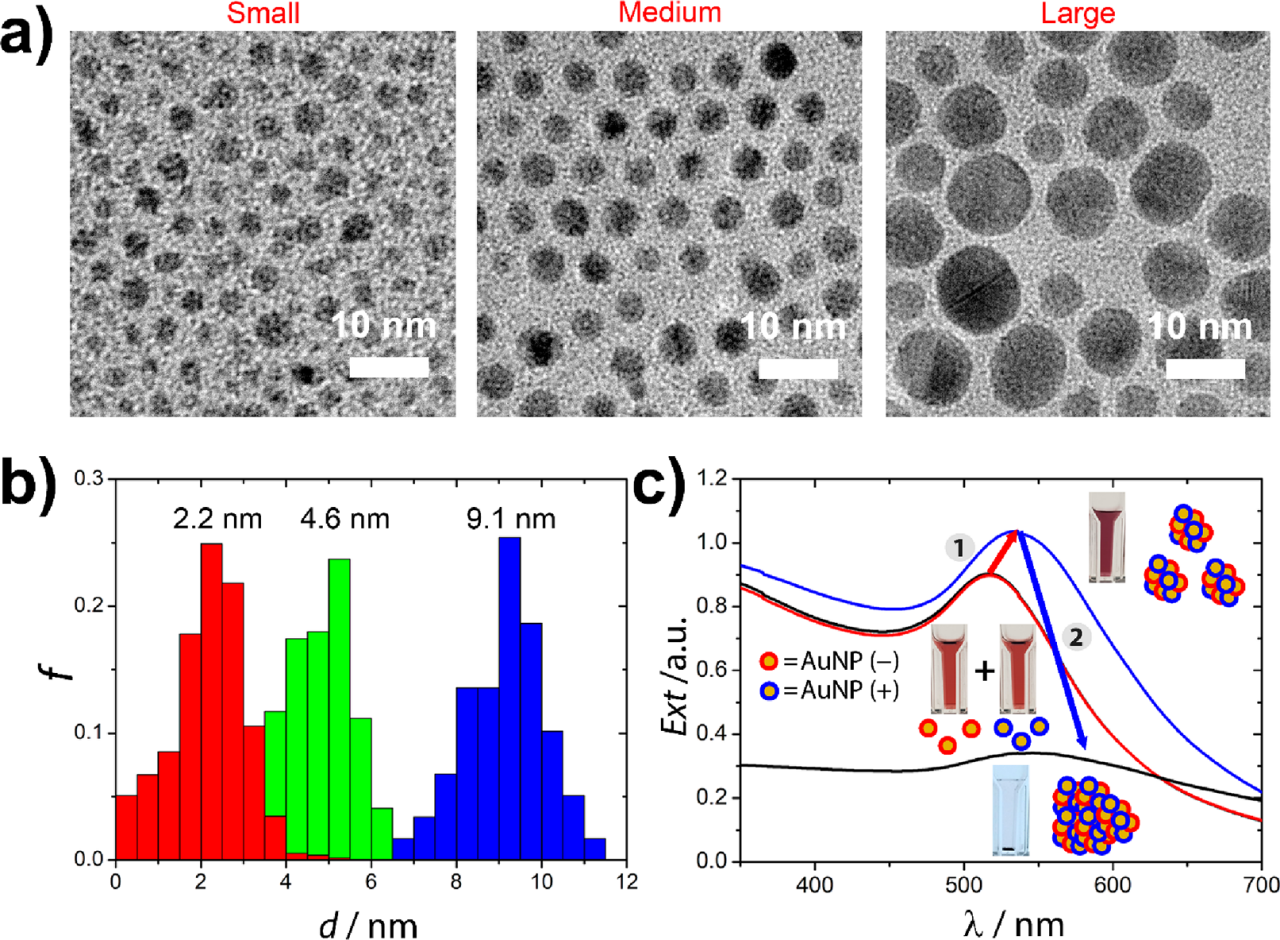
(a) TEM micrographs and (b) size distribution of the AuNPs used in the precipitation experiments of the oppositely charged NPs. (c) The precipitation process of the oppositely charged AuNPs consists of two consecutive steps: (1) aggregation of NPs into clusters manifested in a red-shift of the spectrum (due to an increase of the particle size) and an increase of the extinction (because the red-shifted mode has a larger extinction cross section) and (2) coagulation of these clusters which sediment from the solution manifested in a decrease of the extinction.

Figure 2 shows the results of the electrostatic precipitation of oppositely charged AuNPs. In all mixing ratios, the aggregation of AuNPs into clusters occurred, and the dispersion stability is partially or completely lost. Further away χ = 0.5 (i.e., from the overall electroneutrality), clusters are still formed but remained in the solution. The former can be inferred from the slight increase in the extinction of the samples measured at λ = 523 nm and the pronounced color change from red to purple (Figure 2a,b). Closer to macroscopic electroneutrality, the generated clusters coagulated and formed larger aggregates that sedimented from the liquid phase of the samples. This process manifests in a significant decrease in the extent of extinction and the appearance of the sediments at the bottom of the cuvettes. It has to be pointed out that the width of the symmetric precipitation “window” near χ = 0.5 depends on the size and the concentration of the solutions (Figure 2b). The size effect can be explained by considering the net colloidal interaction between the spheres. In the present system, both the electric double layer and van der Waals interactions promote particle aggregation as a result of attraction. It has to be emphasized, however, that the van der Waals interaction scales with the second order of the radius; hence, a small increase in particle size can result in larger attraction, especially if one considers that the Hamaker coefficient for the gold/water/gold system is a factor larger than usual (∼2.5 × 10^–19^ J) due to the polarizability of the particles.^40^ Thus, the extent of colloidal stability of nanoparticle clusters consisting of the same number of NPs depends on the size of the building blocks: the smaller the NP size, the larger the cluster stability. This dependency is further explained via our model calculations (*vide infra*). At a lower concentration of the AuNPs (0.26 mM), the size of the formed clusters was smaller, improving the stability of the small aggregates in a larger mixing ratio range (the precipitation window shrank (Figures 2c and S2).

**Figure 2:**
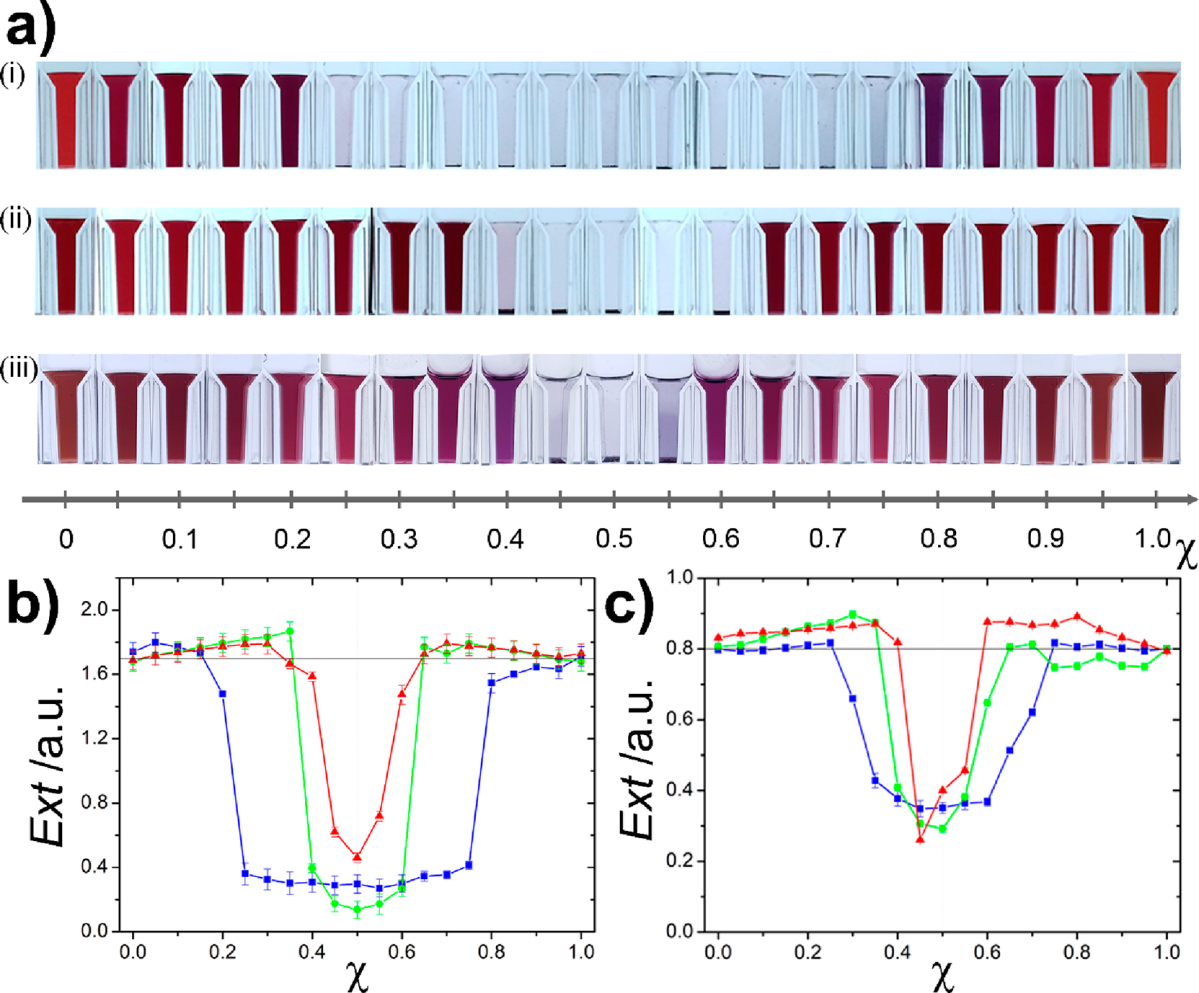
(a) Photographs of the mixtures of the oppositely charged AuNPs applying various mixing ratios after 1 h, starting the experiments using (i) large, (ii) medium, and (iii) small AuNPs with the concentration of 0.56 mM (in terms of gold atoms). (b) Extinction of oppositely charged AuNP mixtures at various mixing ratios after 1 h starting the experiments using AuNPs with the concentration of (b) 0.56 mM and (c) 0.26 mM (measured at λ = 523 nm). The red, green, and blue colors correspond to the small, medium, and large NPs, respectively.

To support the experimental observations, we performed DLS and zeta potential measurements (Figures 3 and S3). In these experiments, the measurements were started immediately after the mixing of solutions of the oppositely charged AuNPs. These measurements confirmed that at χ = 0.5, the precipitation process is the fastest because in this case the formed clusters have the largest size with close to zero zeta potential. Farther from the overall electroneutrality, the size of the clusters decreased and approached the average size of ∼100 nm, where clusters were stable in the water phase as their zeta potential magnitude was ∼40 mV. Interestingly, in small mixing ratios (mixing ratio below 0.3 and above 0.7) the magnitude of the zeta potential slightly increased, which is more expressed in the case of small NPs. This effect can be explained based on the heteroaggregation in the presence of large excess of one of the components: the minority components are effectively shielded by the majority particles, preventing excessive aggregation and consequent sedimentation. Simultaneously, in the same mixing ratio range, the size increases compared to the free particles, confirming heteroaggregation, but remains fairly constant and increases further only as the mixing ratio approaches the value of 0.5. This indicates that a certain limit in terms of the cluster size must be exceeded to observe the overall loss of colloidal stability in the solutions.

**Figure 3:**
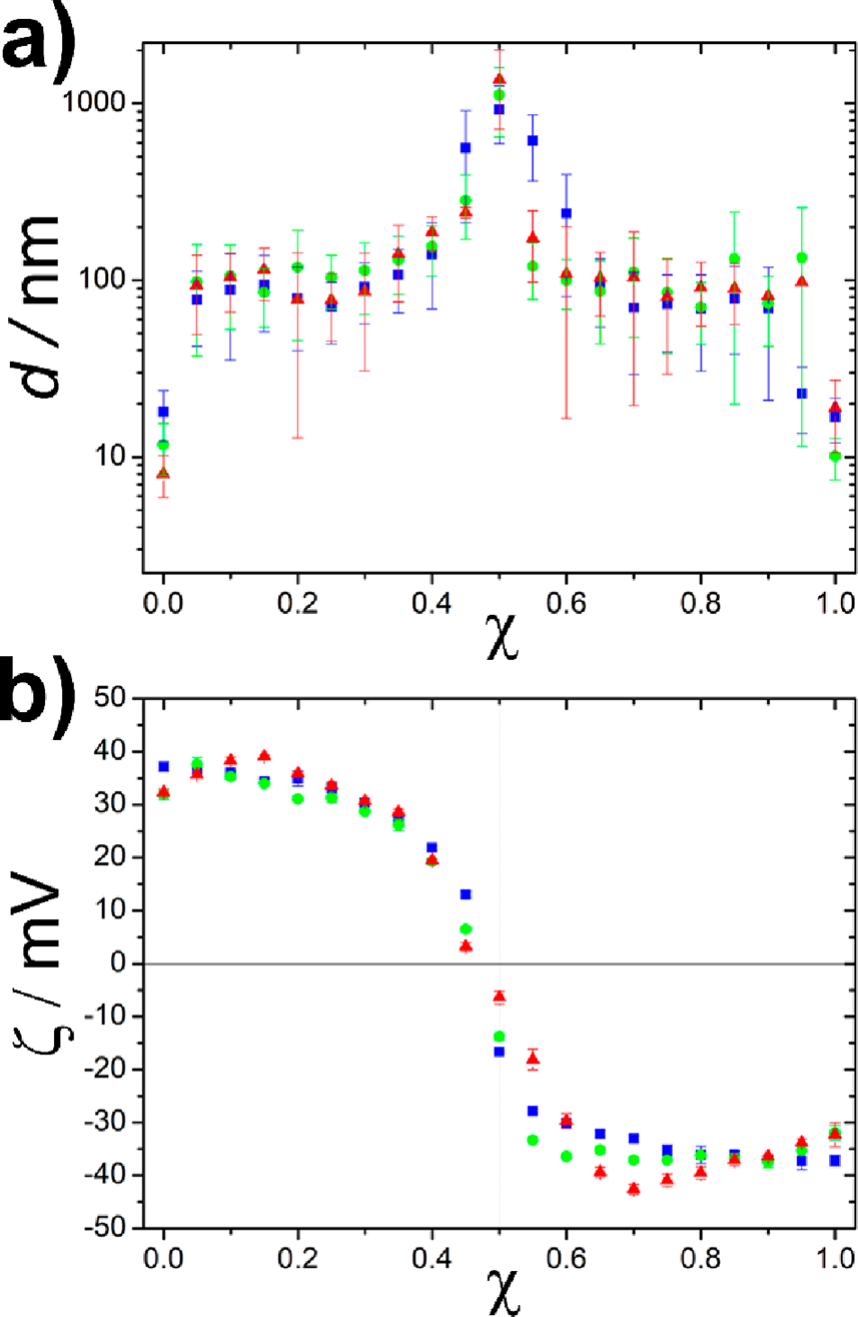
Dependence of (a) the hydrodynamic size (measured by DLS and calculated as an average of the population) and (b) the zeta potential of the clusters formed in the interaction of the oppositely charged NPs on the mixing ratio (χ). The red, green, and blue colors correspond to the small, medium, and large NPs, respectively. The concentration of the solutions of oppositely charged AuNPs was 0.75 mM.

Whereas the observations above are restricted to the behavior right after mixing, the clustering was further investigated for 1 h to create a connection between the instantaneous behavior of the system and the dispersion stability-related sedimentation as a function of time (Figure 4). As heteroaggregation and sedimentation might proceed simultaneously, different quantities have been monitored both during DLS and spectroscopy measurements. Figure 4a shows the intensity-based size evolution of the medium sample at a composition (χ = 0.6), which is inside the precipitation window (see Figure 2a(ii)). It is clear that the initial ca. 100 nm cluster size mentioned earlier right after mixing further increased up to the micrometer range after 1 h. In contrast, for a sample outside the precipitation window (χ = 0.7) the cluster size remains constant at around 100 nm (Figure S4). At the same time, the count rate measured during this 1 h time period starts high and decreases monotonically in the case of χ = 0.6, indicating rapid aggregation followed by steady sedimentation. In contrast, at χ = 0.7 a small count rate increase in the first 10 min shows an ever slowing aggregation leading to stable clusters. This is corroborated by the spectral data of the same systems. The plasmon shift (Figure 4c) changes rapidly within our time resolution (1 min), and the peak position remains almost constant for χ = 0.7, whereas a more pronounced shift is found for χ = 0.6 with similar kinetics as observed for the size change in Figure 4a. The extinction measured at 400 nm related to the Au^0^ content in the light path (Figure 4d) confirms the continuous removal of the particles because of sedimentation when the composition is inside the precipitation window (χ = 0.6), whereas clearly higher dispersion stability is found for the χ = 0.7 composition.

**Figure 4:**
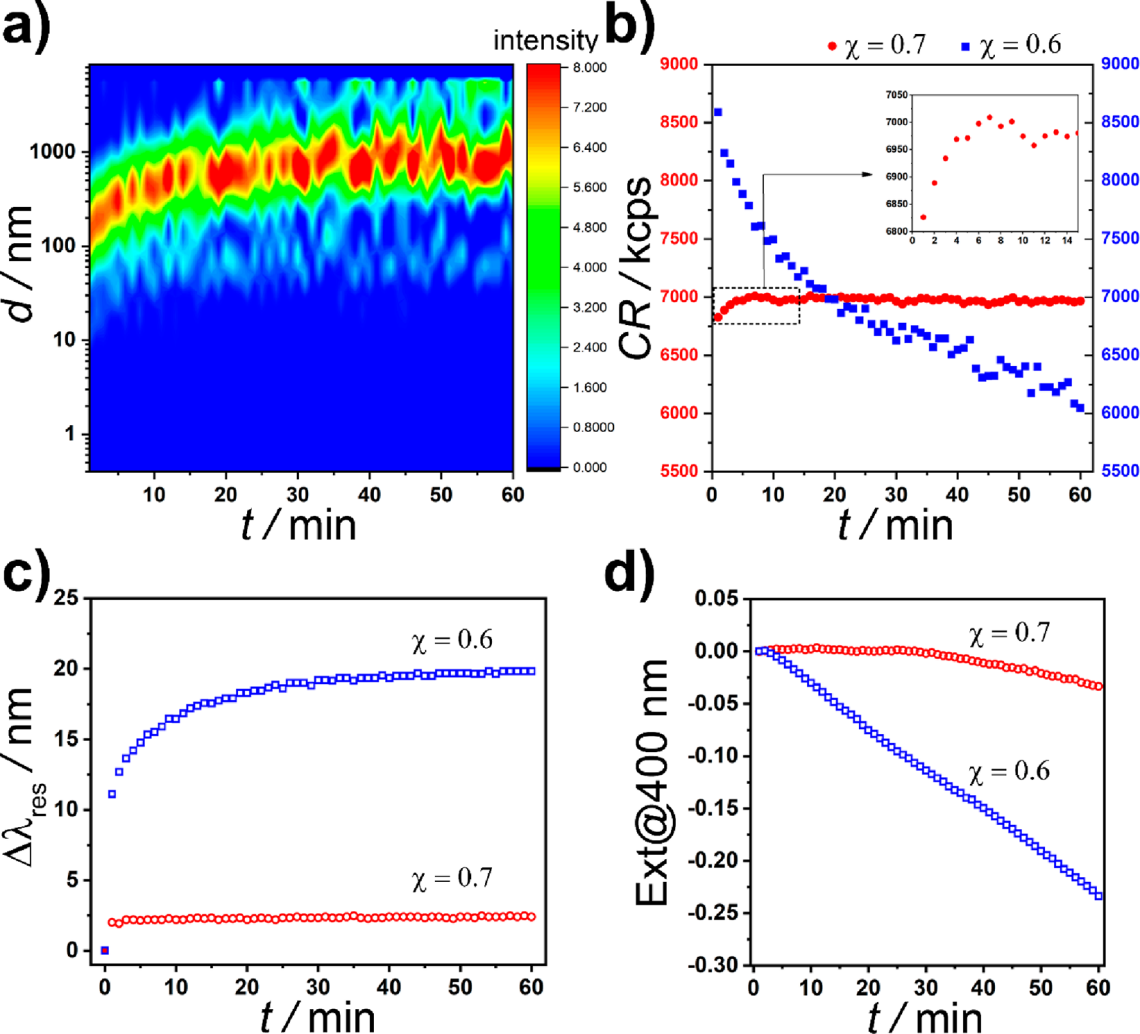
Time-dependent parameters of the medium-sized (4.6 nm) system corresponding to compositions inside (χ = 0.6) and outside (χ = 0.7) of the sedimentation window. The intensity size distribution showing the inside case (a) and overall count rates (b) have been obtained from dynamic light scattering measurements, while the plasmon peak position shift (c) and extinction measured at 400 nm have been extracted from the optical spectra (d).

The same was found for the other two particle sizes (Figures S5 and S6); when χ lies outside the precipitation window, a stable cluster size of around 100 nm is obtained (Figure S4). Inside the window, on the other hand, larger aggregates are formed, leading to sedimentation (for the small-sized sample, the cluster size remains fairly constant though), and the corresponding plasmon peak shifts scale with the particle size as expected (larger particles provide larger shifts). At the same time, the count rate and extinction values are consistent in effectively capturing the sample sedimentation.

To further support the experimental observations, we can construct a mathematical model. The measurements have shown that even if the sample composition is not fulfilling the overall electroneutrality (that is, χ differs from 0.5), clusters are still formed, and the mobility is determined by the charge of the excess particle type. Accordingly, in our simplified model, we assume that at any composition in the internal part of the aggregates the charges are balanced, i.e., the numbers of the oppositely charged NPs are the same, and they form a face-centered cubic crystal lattice. The surface of the aggregates is, however, completely covered by the excess, like charged NPs, which generate a net charge for them. In our model, if the radius of the aggregate (*R*) reaches a critical value (*R*_c_), the formed aggregates sediment from the solution.

Because the lattice constant of a face-centered cubic (FCC) grid is  and it contains two particles, the unit cell area of a single NP on the cluster surface (composed of the excess particles) can be expressed as
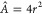
1where *r* is the radius of the NPs. As the volume fraction in the lattice is given by , the volume taken by a single NP in the aggregate is
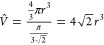
2The volume and surface area of the aggregates can be expressed as
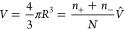
3
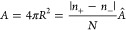
4where *n*_+_ and *n*_–_ are the initial numbers of the positively and negatively charged AuNPs in the sample, respectively, and *N* is the number of aggregates in the sample. In the equations (*n*_+_ + *n*_–_)/*N* gives the number of particles the clusters consist of while |*n*_+_ – *n*_–_|/*N* is the excess on the surface of the aggregates. It has to be emphasized that the cluster surface is covered by the excess particles; hence, their number can be used to derive the surface area of the aggregate. The radius of the cluster (*R*) and the number of the aggregates (*N*) can be expressed from eqs 3 and 4 combining with eqs 1 and 2
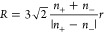
5
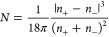
6Analyzing the results of this model (eqs 5 and 6, Figure 5), one can conclude the following findings. The size of the aggregates increases with the size of the NPs (eq 5); thus, in the case of larger NPs the critical radius for sedimentation is reached faster (i.e., the precipitation window is wider for larger NPs). The radius of the aggregates is proportional to the amount (concentration) of the oppositely charged NPs (eq 5; i.e., the precipitation window is wider at higher concentrations of NPs). These two conclusions are in good accordance with the experimental results. The model also implies that the number (concentration) of aggregates does not depend on the size of the NPs (eq 6). Additionally, by increasing the number of the oppositely charged NPs, the number of aggregates decreases in the samples. It should be noted that this model has a singularity at *n*_+_ = *n*_–_, and if *n*_+_ approaches *n*_–_ (or vice versa), the size and number of the aggregates go to infinity and zero (i.e., having only one large aggregate at *n*_+_ = *n*_–_). The model calculations also point out the governing role of the particle size in the cluster stability at different mixing ratios: while the concentration of the clusters does not change upon increasing the size of the building blocks, the dimension of the clusters dictates the stability of the colloidal solutions. The observed mixing-ratio-dependent instability, thus, is a consequence of the increasing interparticle attraction upon increasing the size of the building blocks within the formed clusters.

**Figure 5:**
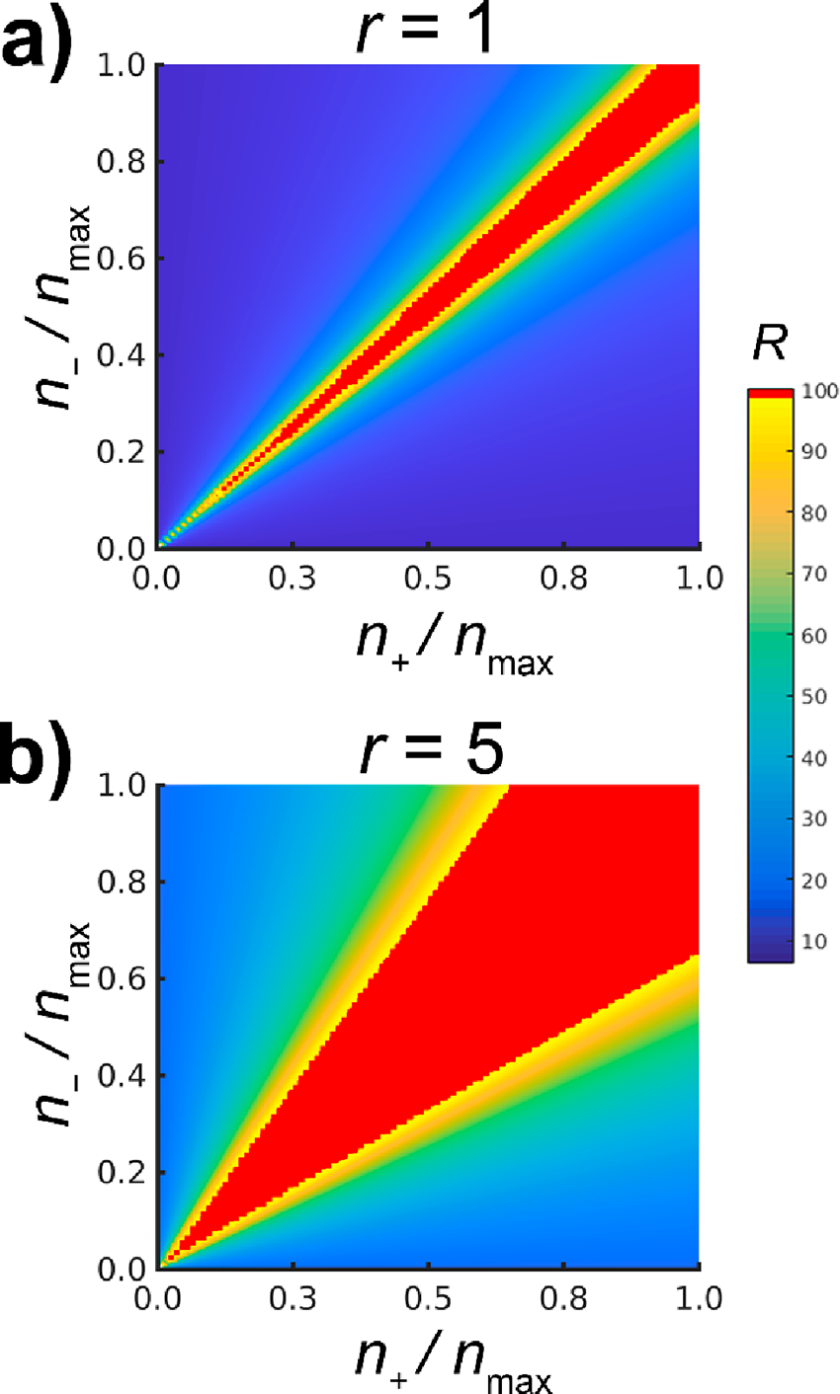
Results of the equilibrium model simulations on the precipitation window using smaller (*r* = 1) and larger (*r* = 5) oppositely charged NPs. The red color corresponds to the size of the clusters that reach the critical size (*R*_c_ = 100) and sediment from the solution. *n*_max_ is the highest initial number of like charged AuNPs.

Here the aim was to develop a minimal model for a qualitative description (with the least possible number of variables) that can highlight the driving force of the phenomena, namely, a simple geometrical arrangement of the NPs. In this approach, to calculate the precipitation window, only the ratio of the oppositely charged NPs and their radii was necessary. To develop such a minimal model, some strong assumptions were required. However, in the Supporting Information, an extended model is presented, which shows that one can observe the same qualitative results with more realistic assumptions introducing further additional parameters, namely, there are free oppositely charged NPs in the system during the precipitation (Figures S7 and S8).

Finally, it is an important issue of how the dispersity of the sample affects the precipitation behavior of the oppositely charged AuNPs. To investigate this effect, polydisperse samples of the oppositely charged AuNPs were created by mixing the like-charged solutions of small, medium, and large AuNPs, keeping the concentration of samples at 0.56 mM in terms of gold atoms in a way that samples contained 10%, 80%, and 10% and 30%, 40%, and 30% small, medium, and large AuNPs in terms of number of NPs, respectively. In the polydisperse samples, the average size of AuNPs increased only by 6% and 15% (from 4.6 to 4.9 and 5.3 nm), respectively. However, the standard deviation was doubled and tripled (from 0.7 to 1.4 and 2.5 nm, Figure S9a–c). The precipitation experiments were performed using these polydisperse samples of oppositely charged AuNPs at various χ values, and the results were compared with those obtained in the case of medium-sized AuNPs (Figures S9d and S10). It can be concluded that the results were similar to that of the medium-sized AuNPs even though the dispersity of the AuNPs samples increased significantly. Based on the photographs and UV–vis measurements of the samples, one can draw the conclusion that mainly the average size of the oppositely charged AuNPs governs the precipitation of NPs, and the dispersity of the sample plays a less significant role. This implies that the size of the major population of the nanoparticles is the crucial parameter in terms of the threshold cluster size, above which the aggregates start to precipitate at the investigated χ resolution.

A detailed understanding of the precipitation of oppositely charged NPs is a key issue not only from the fundamental point of view but also for designing and generating nanostructured materials for various applications. In this study, we present that the oppositely charged and like-sized AuNPs precipitate not only at the point of electroneutrality in the solutions. Based on our findings, the empirical law of precipitation can be revised by the fact that the precipitation process can also occur near the point of electroneutrality. We observed that in all cases when the oppositely charged AuNPs were mixed, irrespective of the ratio, stable clusters were formed with the size of ∼100 nm. Near the point of electroneutrality, these partially stabilized clusters could aggregate and sediment from the solution. We also showed that the precipitation behavior depends rather on the average size of the NPs than the dispersity of the samples. This knowledge can help in the engineering and design of nanostructured and hierarchical materials comprising oppositely charged particles with sizes ranging from nano- to micrometers.

## Supporting Information Available

Synthesis of gold nanoparticles and description of the measurements, description of the extended mathematical model, and Figures S1–S10 showing the calculated overall potentials, the precipitation of oppositely charged gold nanoparticles with the concentration of 0.26 mM; results of the extended mathematical model, and the effect of the dispersity of the sample on the precipitation window (PDF)
Transparent Peer Review report available (PDF)
