# Supplementary material for: Oppositely Charged Nanoparticles Precipitate Not Only at the Point of Overall Electroneutrality
Source: J Phys Chem Lett. 2023 Oct 2;14(40):9003–10. doi: 10.1021/acs.jpclett.3c01857 (PMC10577771; doi:10.1021/acs.jpclett.3c01857)
Supplement: Supplementary file 1 — jz3c01857_si_001.pdf [file jz3c01857_si_001.pdf]

Supporting Information

Oppositely Charged Nanoparticles Precipitate Not Only at the Point of Overall Electroneutrality

Masaki Itatani,[a] Gábor Holló,[b,c] Dániel Zámbó,[d] Hideyuki Nakanishi,[e] András Deák,[d*] István Lagzi,[a,b*]

[a]Department of Physics, Institute of Physics, Budapest University of Technology and Economics, Budapest H-1111, Műegyetem rkp. 3, Hungary

[b]ELKH-BME Condensed Matter Research Group, Budapest H-1111, Műegyetem rkp. 3, Hungary

[c]Department of Fundamental Microbiology, University of Lausanne, Biophore Building, CH-1015, Lausanne, Switzerland

[d]Institute of Technical Physics and Materials Science, Centre for Energy Research, Konkoly-Thege út 29-33, Budapest H-1120, Hungary

[e]Department of Macromolecular Science and Engineering, Graduate School of Science and Technology, Kyoto Institute of Technology, Matsugasaki, Sakyo-ku, Kyoto 606-8585, Japan

Experimental

Synthesis of oppositely charged nanoparticles

We used oppositely charged AuNPs that were synthesized using a modified literature procedure and had three different sizes with average sizes of 2.2, 4.6, and 9.1 nm, respectively.[1] The generated NPs were functionalized with either positively charged (11-mercaptoundecyl)-*N*,*N*,*N*-trimethylammonium bromide (TMA, Sigma-Aldrich) or negatively charged mercaptoundecanoic acid (MUA, Sigma-Aldrich). The pH of both solutions (positively and negatively charged AuNPs) was adjusted to 11.0 with tetramethylammonium hydroxide (Sigma-Aldrich) to ensure that in the precipitation experiments the carboxyl groups on NPs remained fully deprotonated (introducing negative charge; pKa = 6-8 on NPs). The TMA groups were positively charged at that pH (since its pKa > 13).

UV-Vis, DLS, and zeta potential measurements

Precipitation experiments with various ratios of positively and negatively charged AuNPs were carried out in a semi-micro plastic (PMMA) cuvette with 1 cm optical path length and 1.4 mL total volume (the volume of the solutions measured was 1.0 mL). At the beginning of experiments, after mixing the solutions of oppositely charged AuNPs, the cuvettes were capped and gently shaken by hands for 5 seconds. In each ten minutes, the samples were reshaken for 5 seconds. After the last shake (50 min), the samples were undisturbed for 10 min, and then the extinction of the samples was measured by UV-Vis. Finally, the photographs were taken of the samples. Zeta potential and DLS measurements were performed in a Malvern Zetasizer NanoZS using disposable capillary folded cells (DTS1070, 700 μL sample volume) and disposable PMMA cuvettes, respectively. All experiments were carried out at room temperature (22.0 ± 0.5 °C). The extinction of the samples was measured by a UV-Vis spectrophotometer (VWR UV-1600PC) at λ = 523 nm which corresponds to the absorption peak of the surface plasmon resonance of sub-10 nm sized AuNPs. Time-dependent optical spectra have been measured using a Shimadzu UV-3600i Plus spectrophotometer. In zeta potential, DLS, and time-dependent UV-Vis measurements the samples were undisturbed in the cuvettes.

Extended mathematical model

The strictest assumptions in the model – described in the text (Equations 3 and 4) – were that every particle takes part in the formation of aggregates (free particles were not present in the system) and the surface of the aggregates was fully covered with the majority particles. These simplified assumptions were modified in the extended model: (i) a composition-dependent function was introduced to describe the ratio of the free particles and (ii) a new parameter (ω) was applied to determine the excess of most particles on the surface of the aggregates. If not every particle took part in the formation of aggregates, then there were remaining free NPs in the solution, and the volume of the aggregates can be calculated similarly to Equation 3:

$$V = \frac{4}{3}\pi R^3 = \frac{\tilde{n}_+ + \tilde{n}_-}{N}\hat{V}, \tag{S1}$$

where $\tilde{n}_+$ and $\tilde{n}_-$ are the numbers of the aggregate forming particles. (The number of the free particles in the solution can be determined as $n_+^{free} = n_+ - \tilde{n}_+$ and $n_-^{free} = n_- - \tilde{n}_-$, respectively. Here $n_+$and n_- are the initial numbers of the oppositely charged NPs). To fine-tune the model to some experimental observation, one can define a function for the aggregated particles: $\tilde{n} = f(n_+, n_-)$. For simplicity, we proposed a function (as an example) with only one parameter, γ, which describes the ratio of the aggregate forming particles when one type of particle is in great excess:

$$\tilde{n}_+ = n_+\left(1 - (1-\gamma)\left(1 - e^{-\frac{n_+ - n_-}{n_-}}\right)\Theta(n_+ - n_-)\right), \tag{S2}$$

$$\tilde{n}_- = n_-\left(1 - (1-\gamma)\left(1 - e^{-\frac{n_- - n_+}{n_+}}\right)\Theta(n_- - n_+)\right). \tag{S3}$$

This function contains the Heaviside step function, which ensures that particles in excess provide the free particles (in other words, all the minority particles are built up in the aggregates). Furthermore, when the ratio of the positively and negatively charged NPs goes to one, the number of free NPs goes to zero. The effect of this function can be seen in Figure S7. If the surface is not covered with the majority of particles, then Equation 4 must be modified:

$$A = 4\pi R^2 = \frac{|\tilde{n}_+ - \tilde{n}_-|}{\omega N}\hat{A}, \tag{S4}$$

where ω represents the excess ratio of the majority particles on the surface (e.g., with $\omega = 0$, the number of the positively and negatively charged particles is the same). With the newly introduced variables Eqs. 5 and 6 in the text can be modified and the extended model can be written as:

$$R = 3\sqrt{2}\omega \frac{\tilde{n}_+ + \tilde{n}_-}{|\tilde{n}_- - \tilde{n}_-|} r, \tag{S5}$$

$$N = \frac{1}{18\pi\omega^3} \frac{|\tilde{n}_+ - \tilde{n}_-|^3}{(\tilde{n}_+ + \tilde{n}_-)^2}. \tag{S6}$$

Figures S7 and S8 show the results of the extended model, and the obtained results qualitatively agree with the results of the simplified model.

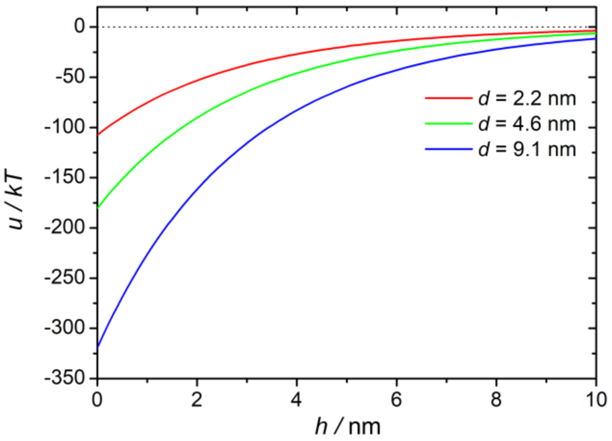

Figure S1 Interaction potentials u (calculated as a sum of the Van der Waals and electrostatic potentials) for oppositely charged and like-sized AuNPs of different sizes. The potentials were calculated based on the potential functions presented in Refs [2,3] with the following parameters: the Hamaker constant ($A = 4\times10^{-19}$ J), radii of the metal cores are 1.1, 2,3 and 4.55 nm, respectively; the thickness of the thiol monolayer on the particles (δ = 1.6 nm), temperature (T = 298 K), the salt concentration (c_s = 0.01 M), the Gibbs energy of ion dissociation in the absence of any external fields ($\Delta G_d = 2.1 \times 10^{-20}$ J), Γ is the surface density of ligands at the NP surface ($\Gamma = 4.7 \times 10^{18}$ m^{-2}), the dielectric constant of the solvent (ε = 80), the mole fraction of counter ions in solution ($x_B = c_s$ /55).

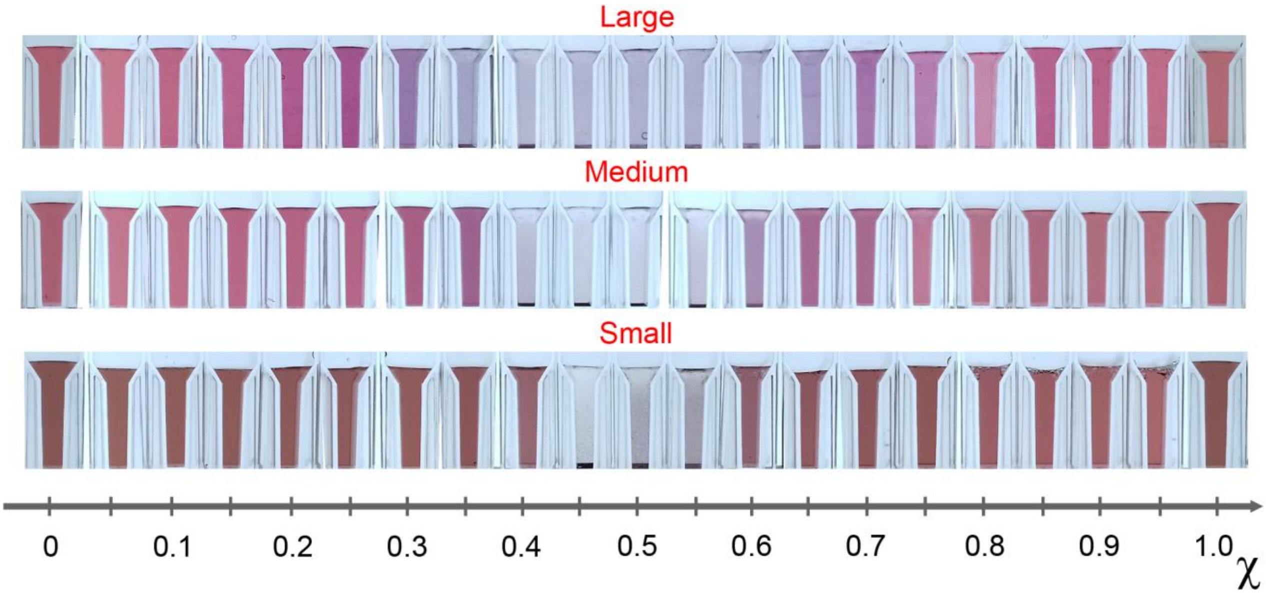

Figure S2 Photographs of the mixtures of the oppositely charged AuNPs using various mixing ratios after 1 h starting the experiments using large (*d* = 9.1 nm), medium (*d* = 4.6 nm), and small (*d* = 2.2 nm) AuNPs with the concentration of 0.26 mM (in terms of gold atoms).

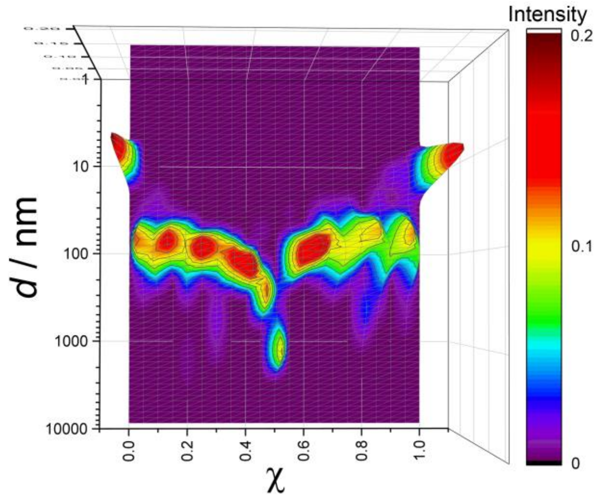

Figure S3 Dependence of the size distribution of the clusters (measured by DLS) formed in the interaction of the medium-sized oppositely charged NPs on the mixing ratio (χ). The concentration of the solutions of the oppositely charged AuNPs was 0.75 mM.

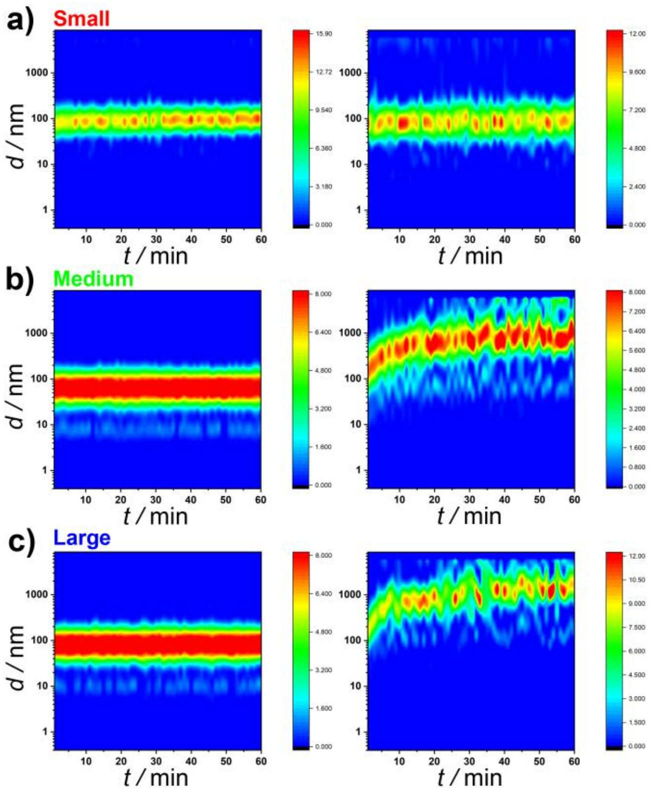

Figure S4 Time-dependent intensity size distributions obtained for the small (2.2 nm - a), medium (4.6 nm - b), and large (9.1 nm - c) particle types outside (left column) and inside (right column) of the sedimentation window.

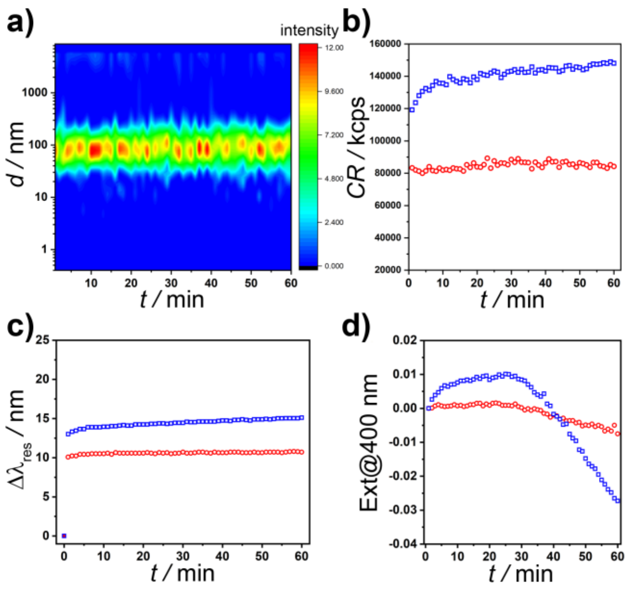

Figure S5 Time-dependent parameters of the small-sized (2.2 nm) system corresponding to compositions inside and outside the sedimentation window. The intensity size distribution showing the inside case (a) and overall count rates (b) have been obtained from dynamic light scattering measurements, while the plasmon peak position shift (c) and extinction measured at 400 nm have been extracted from the optical spectra (d). Blue and red symbols in panels a, b, and c correspond to the inside and outside the sedimentation window, respectively.

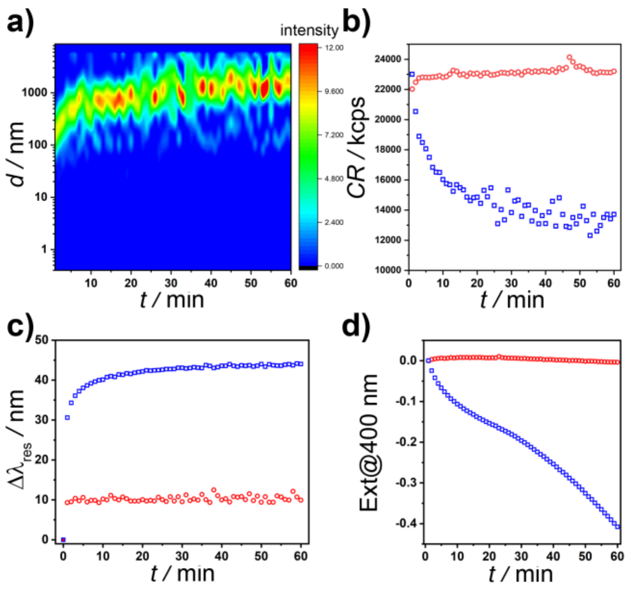

Figure S6 Time-dependent parameters of the large-sized (9.1 nm) system corresponding to compositions inside and outside the sedimentation window. The intensity size distribution showing the inside case (a) and overall count rates (b) have been obtained from dynamic light scattering measurements, while the plasmon peak position shift (c) and extinction measured at 400 nm have been extracted from the optical spectra (d). Blue and red symbols in panels a, b, and c correspond to the inside and outside the sedimentation window, respectively.

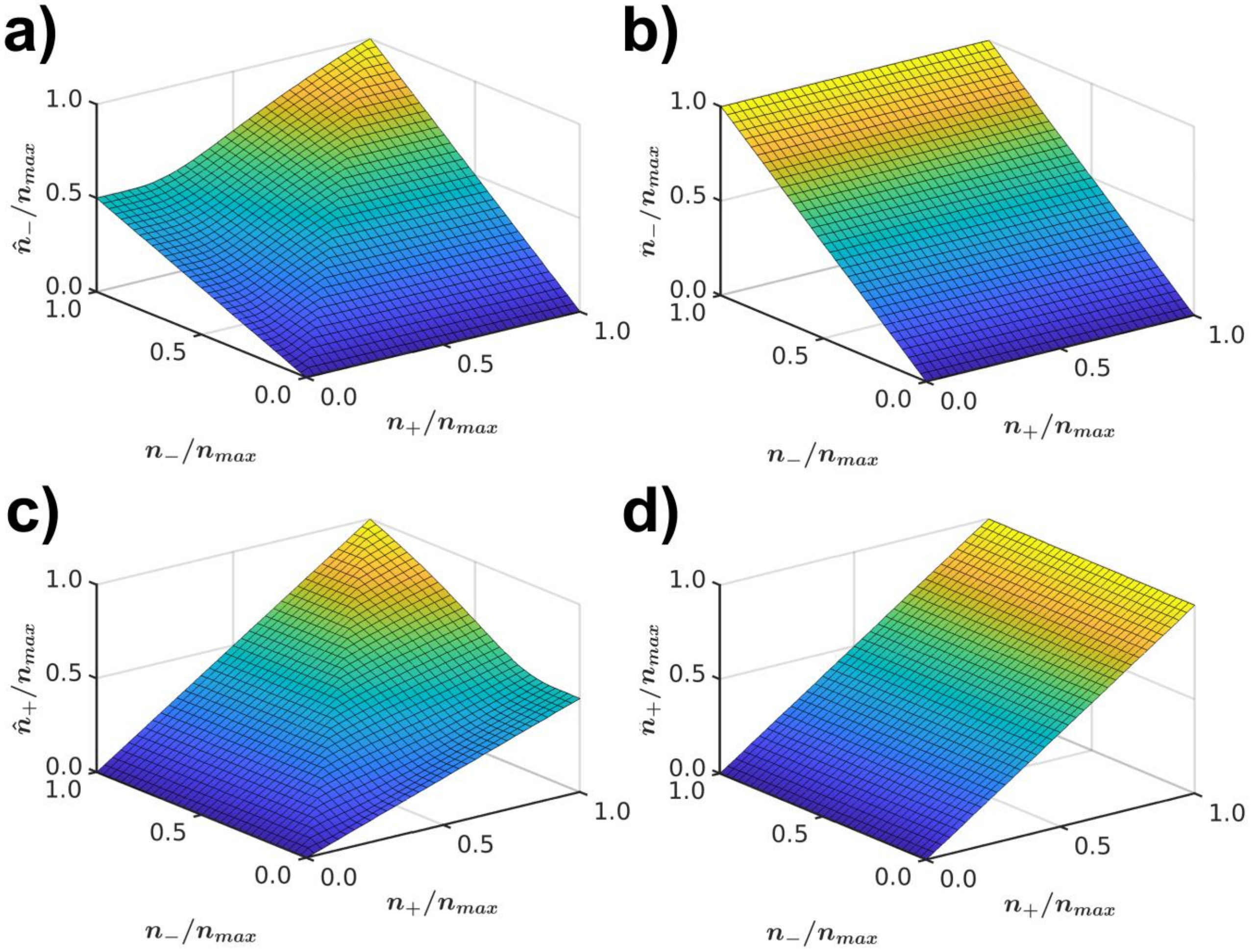

Figure S7 Dependence of the number of the particles built up in the aggregates ($\tilde{n}_+$ and $\tilde{n}_-$) on the excess ratio of the majority particles on the surface (ω). $\omega = 0.5$ (a, c) and $\omega = 1$ (b, d). $\omega = 1$ corresponds to the simplified model discussed in the text. n_{max} is the highest initial number of like-charged AuNPs used in the simulations.

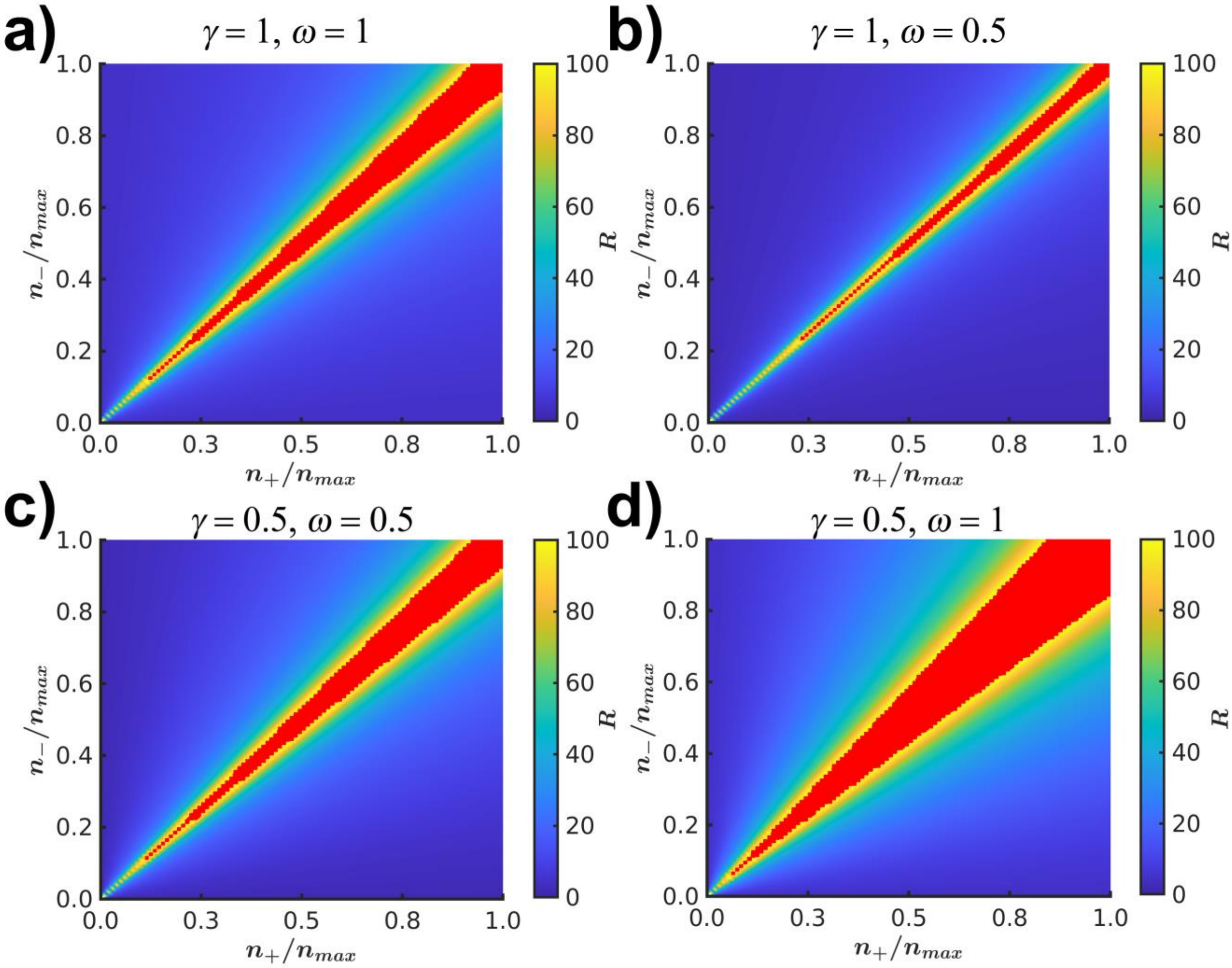

Figure S8 Results of the equilibrium model simulations on the precipitation window using various parameters of $\gamma$and ω. The size of the oppositely charged NPs was $r = 1$. The red color corresponds to the size of the clusters that reach the critical size ($R_c = 100$) and sediment from the solution. $n_{\max}$ is the highest initial number of like-charged AuNPs used in the simulations.

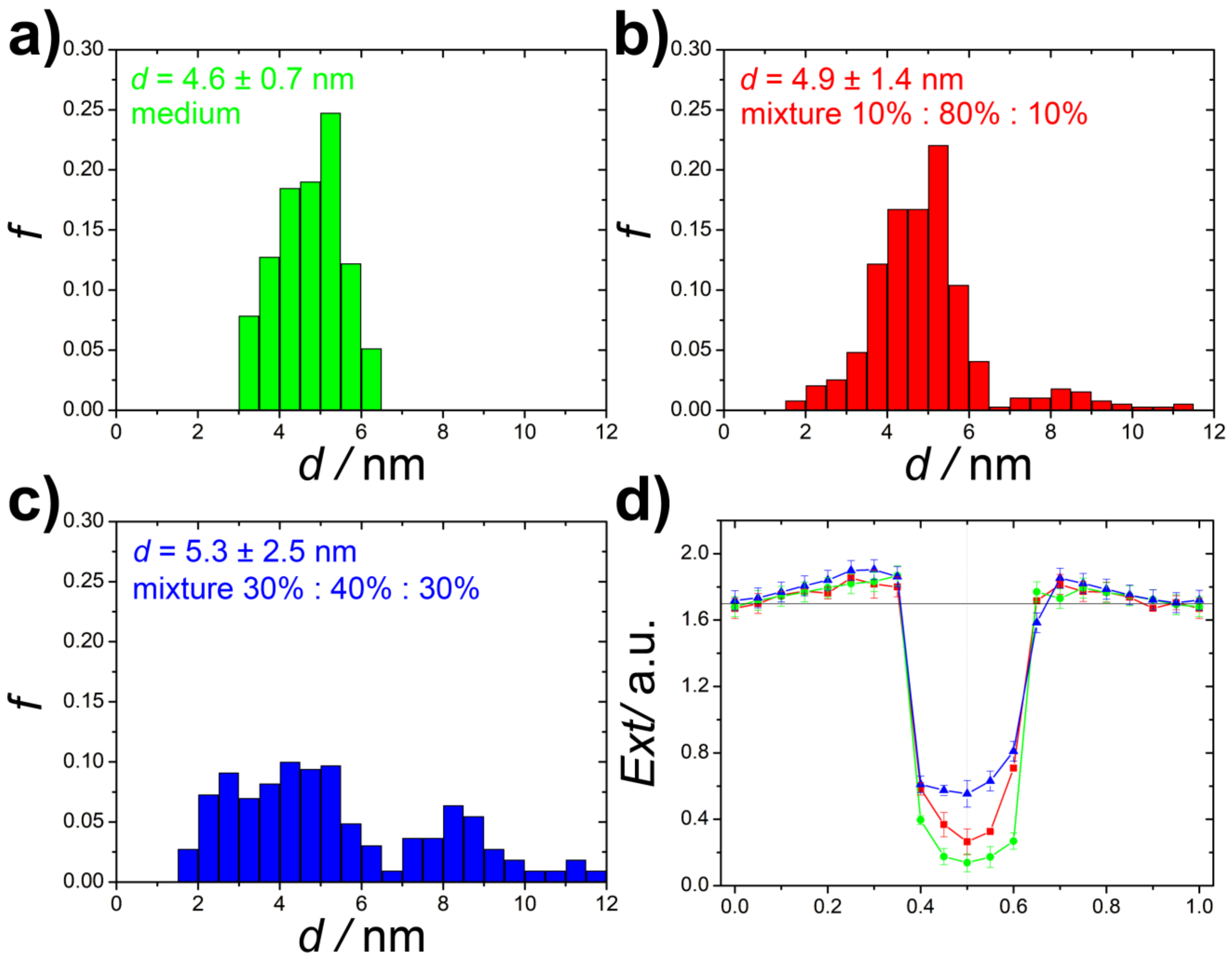

Figure S9 Investigation of the effect of the dispersity of the samples on the precipitation behavior of oppositely charged AuNPs. (a) Size distribution of the medium-sized AuNPs (green), (b) size distribution of a polydisperse sample of AuNPs containing small (10%), medium (80%), and large (10%), and (c) size distribution of a polydisperse sample of AuNPs containing small (30%), medium (40%), and large (30%) AuNPs in terms of the number of NPs used in the precipitation experiments of the oppositely charged NPs. (b) Extinction of oppositely charged AuNP mixtures at various mixing ratios using medium-sized and polydisperse samples after 1 h starting the experiments using AuNPs with the concentration of 0.56 mM (measured at λ = 523 nm).

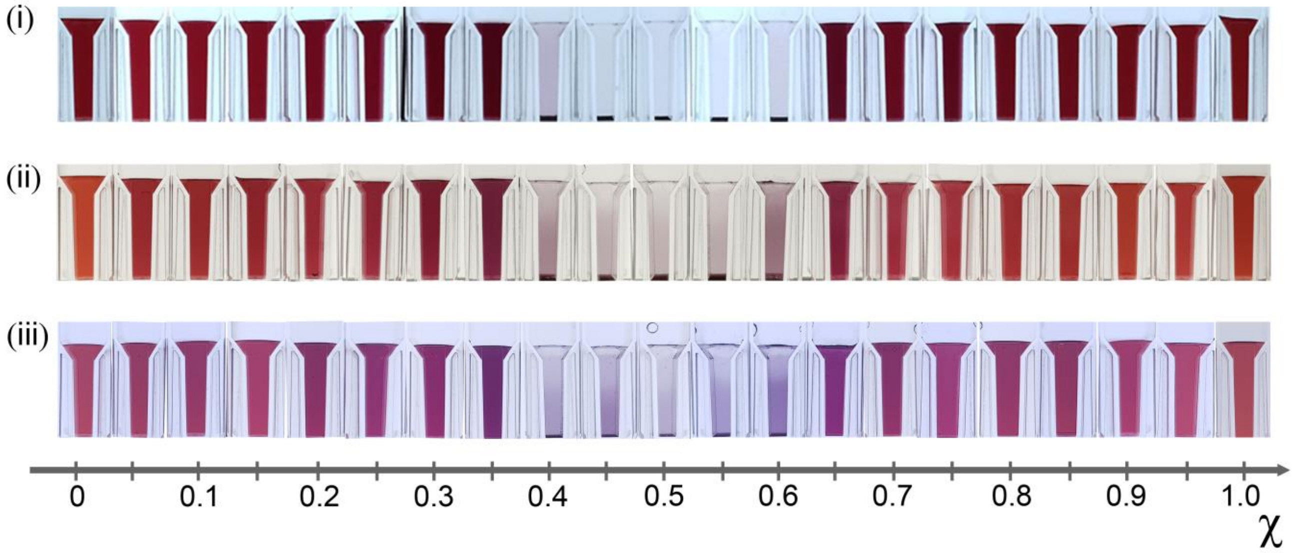

Figure S10 Photographs of the mixtures of the oppositely charged AuNPs applying various mixing ratios after 1 h starting the experiments using (i) medium-sized AuNPs, (ii) a polydisperse sample of AuNPs containing small (10%), medium (80%) and large (10%), and (iii) a polydisperse sample of AuNPs containing small (30%), medium (40%) and large (30%) AuNPs in terms of number of NPs with the concentration of 0.56 mM (in terms of gold atoms).

References

(1) Peter, B.; Lagzi, I.; Teraji, S.; Nakanishi, H.; Cervenak, L.; Zámbó, D.; Deák, A.; Molnár, K.; Truszka, M.; Szekacs, I.; Horvath, R. Interaction of Positively Charged Gold Nanoparticles with Cancer Cells Monitored by an in Situ Label-Free Optical Biosensor and Transmission Electron Microscopy. *ACS Appl. Mater. Interfaces* **2018**, *10* (32), 26841–26850.

(2) Bishop, K. J. M.; Kowalczyk, B.; Grzybowski, B. A. Precipitation of Oppositely Charged Nanoparticles by Dilution and/or Temperature Increase. *J. Phys. Chem. B* **2009**, *113* (5), 1413–1417.

(3) Nakanishi, H.; Deák, A.; Hólló, G.; Lagzi, I. Existence of a Precipitation Threshold in the Electrostatic Precipitation of Oppositely Charged Nanoparticles. *Angew. Chem. Int. Ed.* **2018**, *57* (49), 16062–16066.
