# Supplementary material for: Oppositely Charged Nanoparticles Precipitate Not Only at the Point of Overall Electroneutrality
Source: J Phys Chem Lett. 2023 Oct 2;14(40):9003–10. doi: 10.1021/acs.jpclett.3c01857 (PMC10577771; doi:10.1021/acs.jpclett.3c01857)
Supplement: Supplementary file 2 — jz3c01857_si_002.pdf [file jz3c01857_si_002.pdf]

jz-2023-01857a.R1

Name: Peer Review Information for "Oppositely Charged Nanoparticles Precipitate Not Only at the Point of Overall Electroneutrality"

First Round of Reviewer Comments

Reviewer: 1

Comments to the Author

Comments to the manuscript #jz-2023-01857a

This manuscript focused on precipitation behavior of charge nanoparticles. Similar to molecular electrolytes, oppositely charged nanoparticles form cluster that grow and finally precipitate under appropriate condition. The spectrum of nanoparticle dispersion, namely color of the solution, changes corresponding to the size of nanoparticles and its clusters. The manuscript carefully observed the necessary condition for the precipitation with varying the size of nanoparticles and ratio of positive and negative charged nanoparticles. When the ratio of positive and negative charged nanoparticles was close to 0.5, precipitation occurred. The critical value of the ratio for the precipitation was further deviated from the value of 0.5 with increase in the particles size. In addition, the observation results tried to be explained using theoretical approach.

The target and approaches are interesting. However, the supported theory and discussion has been not yet enough. It should be discussed in detail and publish on more specific journals, for example, Langmuir and J. Phys. Chem. C. The detail comments and questions are listed below.

Q1. For Fig. 2: The □ = 0.5 for Fig. 2a-iii looks like higher value of Ext rather than □ = 0.55 and 0.45. Please explain the reason why this phenomenon occurred. In addition, Fig. 2b does not show it. What happen?

Q2. For theoretical approach, what is “the unit cell area of a single NP”? I can understand the lattice constant of FCC crystal lattice is $2\sqrt{2}r$. So, the area of unit cell of FCC is $8r^2$. It includes 2 particles, so a single NP will cover $4r^2$. Probably, it is due to the authors did not consider crystal but “aggregate”. The assumption must be clarified in the main text.

Q3. The suggested model, equilibrium model which ignores the dynamics of aggregation process, is unique and interesting. However, the discussion includes a big mistake. Based on the equation 5, the authors concluded that the size of aggregates is proportional to the particle radius (r). Here, we have to

consider about the radius dependency of the number $n_{\pm}$. In the experiments, the total amount of Au was kept to constant. Therefore, the number of particles $n_{\pm}$ is proportional to r^{-3}. Thus, the size of aggregates will be proportional to r^{-2}. This result does not agree with experimental observations. I think that the suggested model is too simple to explain the complex aggregation process.

Q4. In the abstract, the authors clearly pointed out that precipitation processes of charged nanoparticles are different from that of ions. I agree. This is very important point of this manuscript. Thus, the authors should clearly describe what is the different point between precipitation of ions and nanoions.

Reviewer: 2

Comments to the Author

The manuscript describes the precipitation behavior of oppositely charged gold nanoparticles (AuNPs). The present study is an important add-on to a series of previous reports by Grzybowski and co-workers. In contrast to earlier studies, the authors report a window rather than a sharp point for the precipitation of oppositely charged AuNPs. The width of the precipitation process depends on the size and concentration of AuNPs. The work is interesting, and deserves a publication in JPCL. The following points should be clarified before publishing the work.

1. Grzybowski and co-workers had already studied the size-effect on the precipitation behavior of oppositely charged AuNPs in the size range of ~3-11 nm (for e.g. J. Am. Chem. Soc. 2006, 128, 15046–15047). It was observed that the precipitation happens only at the point of electroneutrality. Surprisingly, the submitted manuscript reports a window for precipitation. This contrasting observation should be supported with detailed reasoning. Are the experimental conditions for titrations and precipitation similar to the previous reports by Grzybowski and co-workers?

2. Details of NP titration and monitoring of the precipitation process should be included. For instance, it is not clear whether the system was kept under stirring or undisturbed, for 1h, after each addition of oppositely charged NPs. All these could play a crucial role in the precipitation process.

3. NPs used in this study are polydisperse. Does the degree of polydispersity have any role in the origin and width of the NP precipitation window? Authors have done precipitation study by mixing three sizes: 80% medium, 10% large and 10% small NPs. Will the outcome be same if the relative composition of the three NP sizes are changed, like: 40 %, 30% & 30%?

Author's Response to Peer Review Comments:

István Lagzi
Associate Professor
Budapest University of Technology and Economics
Institute of Physics

H-1111, Budafoki út 8.
Budapest, Hungary
Tel: +36 1463-1341
WWW: nimbus.elte.hu/~lagzi
Email: lagzi.istvan.laszlo@ttk.bme.hu

September 01, 2023

Prof. Senior Editor

Dear Professor,

Thank you for sending us the very helpful comments on our manuscript entitled „***Oppositely Charged Nanoparticles Precipitate Not Only at the Point of Overall Electroneutrality***”. In the following, we provide our point-by-point answers to the specific queries. For clarity, the *Reviewers' comments and questions are formatted in italic*.

Manuscript Formatting Request - Non-scientific changes

"1) TOC Graphic: Please resize the TOC graphic per journal guidelines (2 in x 2 in) and move to the correct position (on the same page as the abstract)."

Author reply: The TOC Graphic has been resized, and it is on the same page as the abstract.

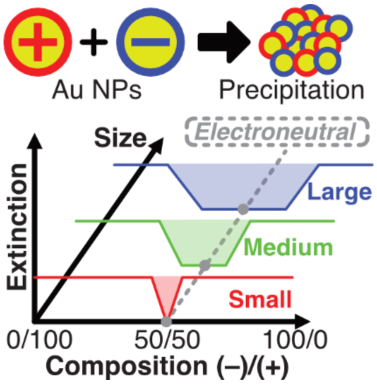

*“2) References: In both the main file and the supporting information, fix the style of all references to use JPCL formatting (check all references carefully). ***JPC Letters reference formatting requires that journal references should contain: () around numbers; author names; article title (titles entirely in title case or entirely in lower case); abbreviated journal title (italicized); year (bolded); volume (italicized); and pages (first-last). Book references should contain author names; book title (in the same pattern); publisher; city; and year. Websites must include date of access.”*

Author reply: The style of all references has been checked.

Reviewer 1

“This manuscript focused on precipitation behavior of charge nanoparticles. Similar to molecular electrolytes, oppositely charged nanoparticles form cluster that grow and finally precipitate under appropriate condition. The spectrum of nanoparticle dispersion, namely color of the solution, changes corresponding to the size of nanoparticles and its clusters. The manuscript carefully observed the necessary condition for the precipitation with varying the size of nanoparticles and ratio of positive and negative charged nanoparticles. When the ratio of positive and negative charged nanoparticles was close to 0.5, precipitation occurred. The critical value of the ratio for the precipitation was further deviated from the value of 0.5 with increase in the particles size. In addition, the observation results tried to be explained using theoretical approach.

The target and approaches are interesting. However, the supported theory and discussion has been not yet enough. It should be discussed in detail and publish on more specific journals, for example, Langmuir and J. Phys. Chem. C. The detail comments and questions are listed below.”

Author reply: We thank the Reviewer for his/her constructive comments.

“Q1. For Fig. 2: The $\chi = 0.5$ for Fig. 2a-iii looks like higher value of Ext rather than $\chi = 0.55$ and 0.45. Please explain the reason why this phenomenon occurred. In addition, Fig. 2b does not show it. What happen.”

Author reply: We thank the Reviewer for this comment. After 1 hour of the experiments, the cuvettes were placed in a sample holder of the UV-Vis spectrophotometer. After measuring all samples, the cuvettes were put before a light pad by hand to take photographs, and a small disturbance during the transfer caused a small redispersion of the sedimented colloids (specialty in the case of small NPs because close to the point of electroneutrality the samples sedimented slower containing smaller aggregates compared to the particles formed in the cases of medium and large NPs). However, based on the issue raised by the Reviewer, the experiments with small NPs have been repeated.

Changes: Page 8. Figure 2 has been updated containing the experimental results using small oppositely charged NPs.

Budapest University of Technology and Economics
Faculty of Natural Sciences ● Institute of Physics
Fizika Tanszék
1111 Budapest, Budafoki út 8. F.épület, Hungary
Phone: +36 1463-2312, Fax: +36 1463 4180
http://dept.phy.bme.hu

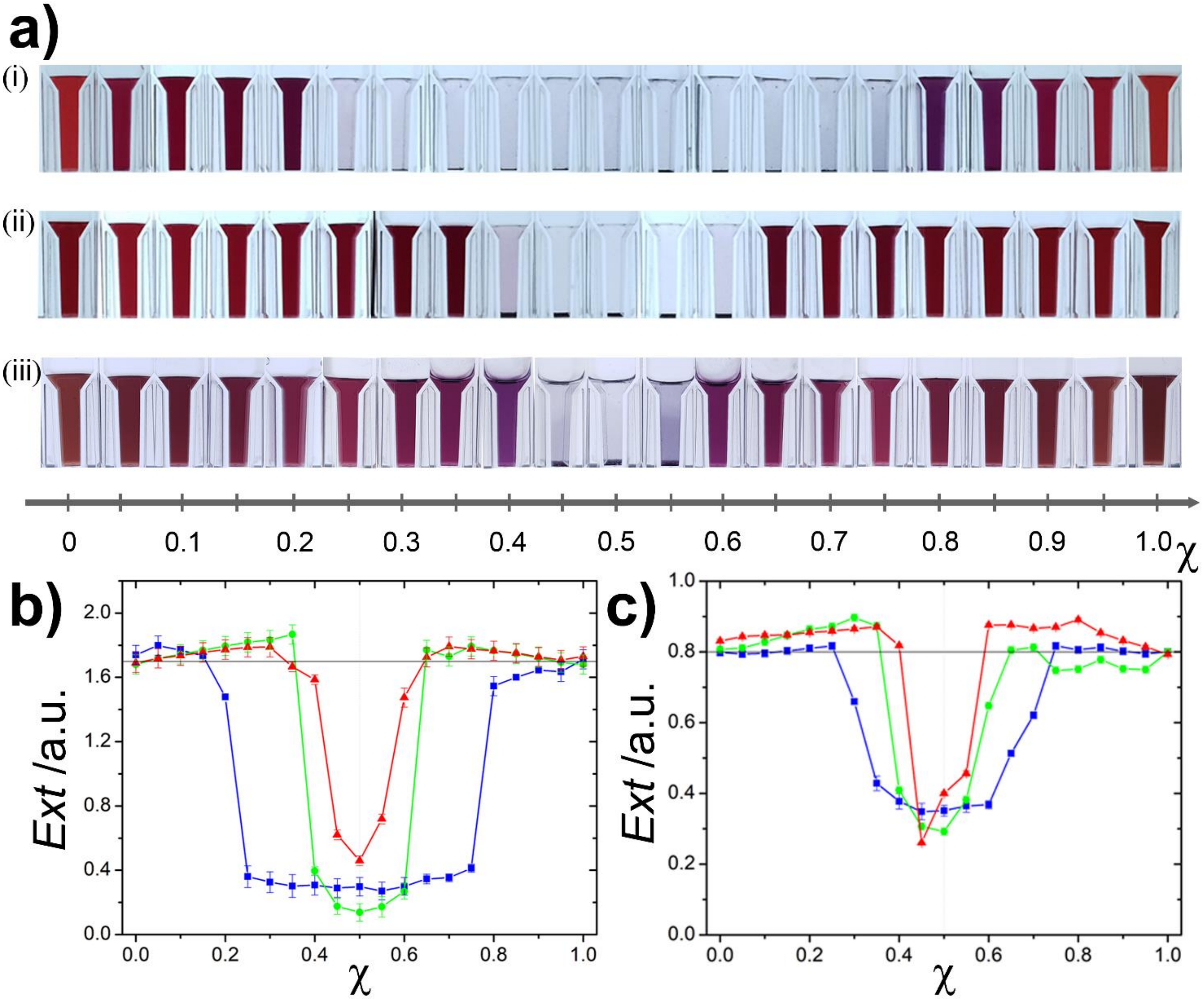

Figure 2 (a) Photographs of the mixtures of the oppositely charged AuNPs applying various mixing ratios after 1 h starting the experiments using (i) large, (ii) medium, and (iii) small AuNPs with the concentration of 0.56 mM (in terms of gold atoms). (b) Extinction of oppositely charged AuNP mixtures at various mixing ratios after 1 h starting the experiments using AuNPs with the concentration of (b) 0.56 mM and (c) 0.26 mM (measured at λ = 523 nm). The red, green, and blue colors correspond to the small, medium, and large NPs, respectively.

"Q2. For theoretical approach, what is "the unit cell area of a single NP"? I can understand the lattice constant of FCC crystal lattice is $2\sqrt{2}r$. So, the area of unit cell of FCC is $8r^2$. It includes 2 particles, so a single NP will cover $4r^2$. Probably, it is due to the authors did not consider crystal but "aggregate". The assumption must be clarified in the main text."

Author reply: This is an important issue. In the model, we hypothesized a two-dimensional

Budapest University of Technology and Economics
Faculty of Natural Sciences ● Institute of Physics
Fizika Tanszék
1111 Budapest, Budafoki út 8. F.épület, Hungary
Phone: +36 1463-2312, Fax: +36 1463 4180
http://dept.phy.bme.hu

hexagonal grid, which consists only of the excess particles and covers the surface of the aggregate. However, based on the Reviewer's comment, we have modified the model calculating the unit surface area from a three-dimensional FCC crystal, which provides a more intuitive interpretation. Therefore, we modified the equations in the manuscript and supplementary information accordingly (Equations 1, 5, 6, S5, and S6). Changing the constant in Equation 1 does not change the essence of our model, but it makes the description more transparent. Therefore, we thank the Reviewer for his/her comment.

Changes: We added the following sentence in the text and modified the following equations in the manuscript and supplementary information. Figure 5 in the manuscript and Figures S7 and S8 in the supplementary information have been updated incorporating the new model simulations.

Pages 13 and 14

Since the lattice constant of a face-centered cubic (FCC) grid is $2\sqrt{2r}$ and it contains two particles, the unit cell area of a single NP on the cluster surface (composed of the excess particles) can be expressed as

$$\hat{A} = 4r^2, \qquad (1)$$

$$R = 3\sqrt{2}\frac{n_+ + n_-}{|n_+ - n_-|}r, \qquad (5)$$

$$N = \frac{1}{18\pi}\frac{|n_+ - n_-|^3}{(n_+ + n_-)^2}. \qquad (6)$$

Page S4

$$R = 3\sqrt{2}\omega\frac{\tilde{n}_+ + \tilde{n}_-}{|\tilde{n}_- - \tilde{n}_-|}r, \qquad (S5)$$

$$N = \frac{1}{18\pi\omega^3}\frac{|\tilde{n}_+ - \tilde{n}_-|^3}{(\tilde{n}_+ + \tilde{n}_-)^2}. \qquad (S6)$$

"Q3. The suggested model, equilibrium model which ignores the dynamics of aggregation process, is unique and interesting. However, the discussion includes a big mistake. Based on the equation 5, the authors concluded that the size of aggregates is proportional to the particle radius (r). Here, we have to consider about the radius dependency of the number $n_\pm$. In the experiments, the total amount of Au was kept to constant. Therefore, the number of particles $n_\pm$ is proportional to r^{-3}. Thus, the size of aggregates will be proportional to r^{-2}. This result does not agree with experimental observations. I think that the suggested model is too simple to explain the complex aggregation process."

Author reply: We thank the Reviewer for this comment. Below, we provide a short derivation supporting our argument. If the number of the gold atoms is constant (and the same) in the stock

Budapest University of Technology and Economics
Faculty of Natural Sciences • Institute of Physics
Fizika Tanszék
1111 Budapest, Budafoki út 8. F.épület, Hungary
Phone: +36 1463-2312, Fax: +36 1463 4180
http://dept.phy.bme.hu

solutions independently of the radius and charge, then the concentration is inversely proportional to r^3 as the reviewer suggested:

$$c_0^+ r^3 = c_0^- r^3 = constant = C,$$

where c_0^+ and c_0^- are the concentrations of the positively and negatively charged nanoparticles (NPs) in the stock solution. The mixture contains $V_\pm$volume of these solutions: $V = V_+ + V_-$ so the number of the NPs can be calculated as $n_\pm = N_{av} V_\pm c_0^\pm$. Replacing these expressions in Equation 5, we get

$$R \approx \frac{n_+ + n_-}{|n_+ - n_-|} r = \frac{N_{av} V_+ c_0^+ + N_{av} V_- c_0^-}{|N_{av} V_+ c_0^+ - N_{av} V_- c_0^-|} r = \frac{V_+ \frac{C}{r^3} + V_- \frac{C}{r^3}}{\left|V_+ \frac{C}{r^3} - V_- \frac{C}{r^3}\right|} r = \frac{V_+ + V_-}{|V_+ - V_-|} r = \frac{V_+/V + V_-/V}{|V_+/V - V_-/V|} r = \frac{x_+ + x_-}{|x_+ - x_-|} r,$$

As it can be seen, $R \approx r$, and the other term contains only the volume ratio ($x_\pm$) of the solution, which is independent of the radius of the NPs. In other words, both the numerator and the denominator have the same dependence of r^{-3}, and they will eventually cancel out each other.

"Q4. In the abstract, the authors clearly pointed out that precipitation processes of charged nanoparticles are different from that of ions. I agree. This is very important point of this manuscript. Thus, the authors should clearly describe what is the different point between precipitation of ions and nanoions."

Author reply: We thank the Reviewer for this issue. Based on the suggestion, we added a short paragraph to the Introduction to highlight the difference between the precipitation of ions and nanoparticles (nanoions).

Changes: Pages 3 and 4. We added the following discussion to the text.

It has been shown earlier that very small (a few nanometers in diameter), oppositely charged nanoparticles show ionic-like behavior during their aggregation, and large, ordered nanoparticle crystals are formed at the point of the overall electroneutrality, where the aggregate formation can be qualitatively described considering the free-energy change associated with the ordered nanoparticle-crystal formation.[33] As for these "nanoions", the electric double-layer interaction has an effective range comparable with the particle diameter. The solubility of the precipitate of nanoions can be considered to be zero, and when one polarity of nanoions is in excess, the formation of core-shell structures can be anticipated, that is the minority component is shielded by the majority of NPs, resulting in small nanoparticle clusters that can remain stable over time without significant sedimentation.[24] For larger particle diameters, similar behavior can be expected, but as their concentration becomes similar, an earlier onset of sedimentation might occur.

(24) Kalsin, A. M.; Kowalczyk, B.; Smoukov, S. K.; Klajn, R.; Grzybowski, B. A. Ionic-like Behavior of Oppositely Charged Nanoparticles. *J. Am. Chem. Soc.* **2006**, *128* (47), 15046–15047.

Budapest University of Technology and Economics
Faculty of Natural Sciences ● Institute of Physics
Fizika Tanszék
1111 Budapest, Budafoki út 8. F.épület, Hungary
Phone: +36 1463-2312, Fax: +36 1463 4180
http://dept.phy.bme.hu

(33) Kalsin, A. M.; Fialkowski, M.; Paszewski, M.; Smoukov, S. K.; Bishop, K. J. M.; Grzybowski, B. A. Electrostatic Self-Assembly of Binary Nanoparticle Crystals with a Diamond-Like Lattice. *Science* **2006**, *312* (5772), 420–424.

Reviewer 2

"The manuscript describes the precipitation behavior of oppositely charged gold nanoparticles (AuNPs). The present study is an important add-on to a series of previous reports by Grzybowski and co-workers. In contrast to earlier studies, the authors report a window rather than a sharp point for the precipitation of oppositely charged AuNPs. The width of the precipitation process depends on the size and concentration of AuNPs. The work is interesting, and deserves a publication in JPCL. The following points should be clarified before publishing the work."

Author reply: We thank the Reviewer for his/her comments and the positive assessment of our work.

"1. Grzybowski and co-workers had already studied the size-effect on the precipitation behavior of oppositely charged AuNPs in the size range of ~3-11 nm (for e.g. J. Am. Chem. Soc. 2006, 128, 15046–15047). It was observed that the precipitation happens only at the point of electroneutrality. Surprisingly, the submitted manuscript reports a window for precipitation. This contrasting observation should be supported with detailed reasoning. Are the experimental conditions for titrations and precipitation similar to the previous reports by Grzybowski and co-workers?"

Author reply: This is a valid point. In the work of Grzybowski and co-workers, the authors titrated the solution of one polarity with the solution of oppositely charged NPs. Below is provided the description of the experimental procedure from the given paper (*J. Am. Chem. Soc. 2006, 128, 15046–15047*)

"In a typical experiment, a stirred solution of NPs C = 0.2-5mM measured in terms of metal atoms, not NPs) of one polarity was titrated with small aliquots of a solution (0.2-5 mM) containing oppositely charged NPs. After each addition, the mixture was allowed to equilibrate for 5-10 min, and its UV-vis spectra and the value of the ξ potential were recorded."

In our study, we followed a different strategy, instead of titration, the prepared samples of oppositely charged NPs were mixed with various volume ratios. After 1 hour, the samples were investigated. It should be noted that the work of Grzybowski and co-workers foresaw the existence of the window for precipitation because the data of zeta potential near the electroneutrality were close to zero indicating unstable colloidal samples. Probably the period of 5-10 min used in the titration experiment was not enough to show fast precipitation near the electroneutrality.

Changes: Page 4. To clarify the difference in the experimental setup used, we added a sentence in the text.

This procedure differed from the method used in previous studies in which the authors titrated the solution of one polarity with the solution of oppositely charged AuNPs.[24,25]

"2. Details of NP titration and monitoring of the precipitation process should be included. For instance, it is not clear whether the system was kept under stirring or undisturbed, for 1h, after each addition of oppositely charged NPs. All these could play a crucial role in the precipitation process."

Author reply: We thank the Reviewer for this important issue. Now, we have provided a robust protocol of our experimental setup in the experimental part of the Supporting Information. In addition, this is a crucial point raised by the Reviewer. In our previous work (Nakanishi *et al.*, Existence of a Precipitation Threshold in the Electrostatic Precipitation of Oppositely Charged Nanoparticles. *Angew. Chem. Int. Ed.* **2018**, *57* (49), 16062–16066), we investigated the effect of the stirring on the precipitation of oppositely charged AuNPs of medium size at the electroneutral condition (see the figure below). We found a trivial finding, namely, that the stirred sample sedimented faster than the undisturbed one. Therefore, it is essential to fix and use the same conditions even if it is a stirred or undisturbed one.

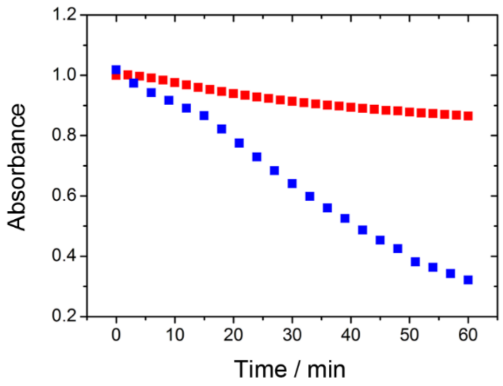

FigureS5 Temporal variation of the absorbance (at λ = 517 nm) of an unstirred (red) and stirred (blue) sample containing a mixture (1:1) of oppositely charged NPs of the same concentrations (c = 0.33 mM, based on gold atoms). (From the publication by Nakanishi et al., Existence of a Precipitation Threshold in the Electrostatic Precipitation of Oppositely Charged Nanoparticles. Angew. Chem. Int. Ed. 2018, 57 (49), 16062–16066.)

Changes: Supporting Information, Page S2

UV-Vis, DLS, and zeta potential measurements

Precipitation experiments with various ratios of positively and negatively charged AuNPs were carried out in a semi-micro plastic (PMMA) cuvette with 1 cm optical path length and 1.4 mL total volume (the volume of the solutions measured was 1.0 mL). At the beginning of experiments, after mixing the solutions of oppositely charged AuNPs, the cuvettes were capped and gently shaken by hands for 5 seconds. In each ten minutes, the samples were reshaken for 5 seconds. After the last shake (50 min), the samples were undisturbed for 10 min, and then the extinction of the samples was measured by UV-Vis. Finally, the photographs were taken of the samples. Zeta potential and DLS

Budapest University of Technology and Economics
Faculty of Natural Sciences • Institute of Physics
Fizika Tanszék

1111 Budapest, Budafoki út 8. F.épület, Hungary
Phone: +36 1463-2312, Fax: +36 1463 4180
http://dept.phy.bme.hu

measurements were performed in a Malvern Zetasizer NanoZS using disposable capillary folded cells (DTS1070, 700 μL sample volume) and disposable PMMA cuvettes, respectively. All experiments were carried out at room temperature (22.0 ± 0.5 °C). The extinction of the samples was measured by a UV-Vis spectrophotometer (VWR UV-1600PC) at λ = 523 nm which corresponds to the absorption peak of the surface plasmon resonance of sub-10 nm sized AuNPs. Time-dependent optical spectra have been measured using a Shimadzu UV-3600i Plus spectrophotometer. In zeta potential, DLS, and time-dependent UV-Vis measurements the samples were undisturbed in the cuvettes.

"3. NPs used in this study are polydisperse. Does the degree of polydispersity have any role in the origin and width of the NP precipitation window? Authors have done precipitation study by mixing three sizes: 80% medium, 10% large and 10% small NPs. Will the outcome be same if the relative composition of the three NP sizes are changed, like: 40 %, 30% & 30%?"

Author reply: We thank the Reviewer for this comment. Indeed, the investigation of the dispersity of the samples is an interesting aspect of this study. Based on the suggestion, we carried out precipitation experiments with a very dispersed sample (40 %, 30%, and 30%). We found that increasing the dispersity of the samples did not significantly affect the window of precipitation.

Changes: We discussed the results of the new experiment in the text (Page 16) and extended Figures S9 and S10 in the supplementary information to incorporate the results.

Finally, it is an important issue how the dispersity of the sample affects the precipitation behavior of the oppositely charged AuNPs. To investigate this effect, polydisperse samples of the oppositely charged AuNPs were created by mixing the like-charged solutions of small, medium, and large AuNPs, keeping the concentration of samples at 0.56 mM in terms of gold atoms in a way that samples contained 10%, 80%, and 10% and 30%, 40%, and 30% of small, medium, and large AuNPs in terms of number of NPs, respectively. In the polydisperse samples, the average size of AuNPs increased only by 6% and 15% (from 4.6 to 4.9 and 5.3 nm), respectively. However, the standard deviation was doubled and tripled (from 0.7 to 1.4 and 2.5 nm, Figure S9a-c). The precipitation experiments were carried out using these polydisperse samples of oppositely charged AuNPs at various χ and compared the results with those obtained in the case of medium-sized AuNPs (Figures S9d and S10). It can be concluded that the results were similar to that of the medium-sized AuNPs even though the dispersity of the AuNPs samples increased significantly. Based on the photographs and UV-Vis measurements of the samples, one can draw the conclusion that mainly the average size of the oppositely charged AuNPs governs the precipitation of NPs and the dispersity of the sample plays a less significant role. This implies that the size of the major population of the nanoparticles is the crucial parameter in terms of the threshold cluster size above which the aggregates start to precipitate at the investigated χ resolution.

Budapest University of Technology and Economics
Faculty of Natural Sciences • Institute of Physics
Fizika Tanszék
1111 Budapest, Budafoki út 8. F.épület, Hungary
Phone: +36 1463-2312, Fax: +36 1463 4180
http://dept.phy.bme.hu

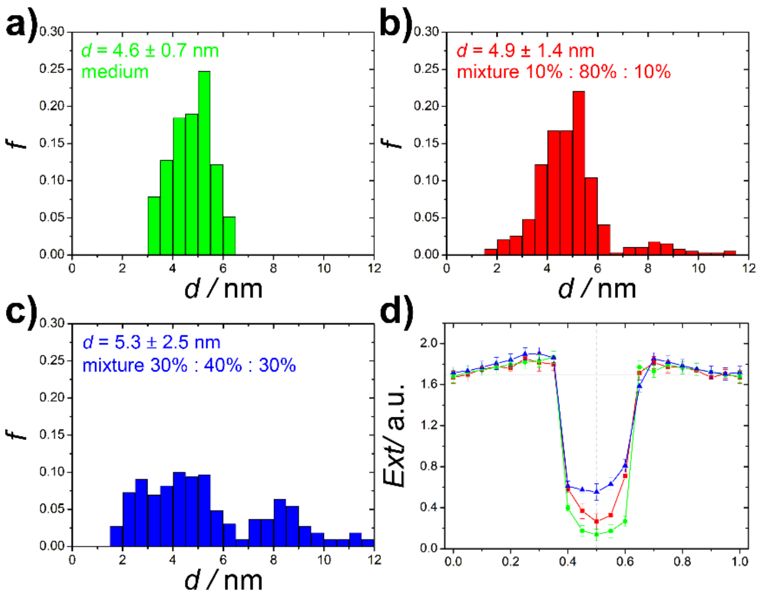

Figure S9 Investigation of the effect of the dispersity of the samples on the precipitation behavior of oppositely charged AuNPs. (a) Size distribution of the medium-sized AuNPs (green), (b) size distribution of a polydisperse sample of AuNPs containing small (10%), medium (80%), and large (10%), and (c) size distribution of a polydisperse sample of AuNPs containing small (30%), medium (40%), and large (30%) AuNPs in terms of the number of NPs used in the precipitation experiments of the oppositely charged NPs. (b) Extinction of oppositely charged AuNP mixtures at various mixing ratios using medium-sized and polydisperse samples after 1 h starting the experiments using AuNPs with the concentration of 0.56 mM (measured at $\lambda = 523$ nm).

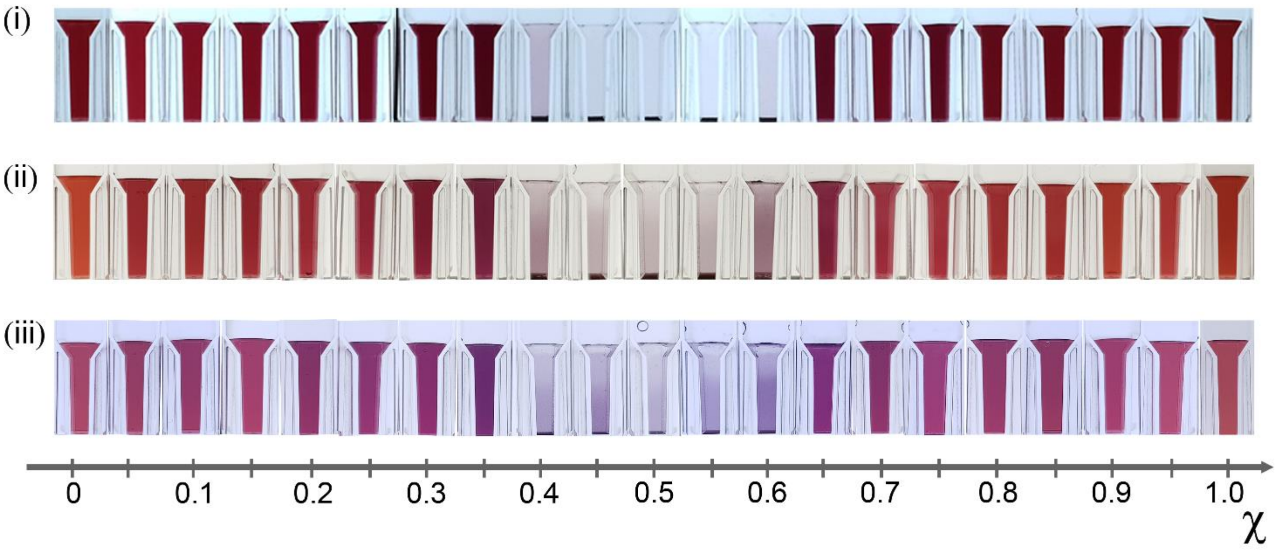

Budapest University of Technology and Economics
Faculty of Natural Sciences • Institute of Physics
Fizika Tanszék
1111 Budapest, Budafoki út 8. F.épület, Hungary
Phone: +36 1463-2312, Fax: +36 1463 4180
http://dept.phy.bme.hu

Figure S10 Photographs of the mixtures of the oppositely charged AuNPs applying various mixing ratios after 1 h starting the experiments using (i) medium-sized AuNPs, (ii) a polydisperse sample of AuNPs containing small (10%), medium (80%) and large (10%), and (iii) a polydisperse sample of AuNPs containing small (30%), medium (40%) and large (30%) AuNPs in terms of number of NPs with the concentration of 0.56 mM (in terms of gold atoms).

This completes our reply. We again thank the Referees for their comments/suggestions and critical reading of our work. We hope that the manuscript is now in an acceptable form.

Sincerely yours,

István Lagzi

Budapest University of Technology and Economics
Faculty of Natural Sciences • Institute of Physics
Fizika Tanszék

1111 Budapest, Budafoki út 8. F.épület, Hungary
Phone: +36 1463-2312, Fax: +36 1463 4180
http://dept.phy.bme.hu
